# Civiale’s Collection of Calculi

**Published:** 1868-01

**Authors:** 


CIVIALE'S COLLECTION OF CALCULI.—Not long before his death, Civiale exhibited to the French Academy, his collection of urinary calculi, from 2700 patients operated on by him during the 43 years of his professional career. In 1600 of the number he had performed his favorite operation of Lithotrity. —*Pacific Med. & Surg. Journal.*

